# Characterizing the Genomic Landscape of Brain Glioma With Circulating Tumor DNA From Tumor *In Situ* Fluid

**DOI:** 10.3389/fonc.2021.584988

**Published:** 2021-03-31

**Authors:** Zhiyuan Sheng, Jinliang Yu, Kaiyuan Deng, Hugo Andrade-Barazarte, Ajmal Zemmar, Sijia Li, Nianxuan Li, Zhaoyue Yan, Zhongcan Chen, Yong Sun, Juha Hernesniemi, Xingyao Bu

**Affiliations:** ^1^ Department of Neurosurgery, Zhengzhou University People’s Hospital, Henan Provincial People’s Hospital, Zhengzhou, China; ^2^ School of Basic Medicine, Henan University of Chinese Medicine, Zhengzhou, China; ^3^ Department of Neurology, The Fifth Affiliated Hospital of Zhengzhou University, Zhengzhou, China

**Keywords:** tumor *in situ* fluid, circulating tumor DNA, glioma, precision medicine, liquid biopsy

## Abstract

Tumor *in situ* fluid (TISF) refers to the fluid at the local surgical cavity. We evaluated the feasibility of TISF-derived circulating tumor DNA (ctDNA) characterizing the genomic landscape for glioma. This retrospective study included TISF and tumor samples from 10 patients with glioma, we extracted cell-free DNA (cfDNA) from the TISF and then performed deep sequencing on that. And we compared genomic alterations between TISF and tumor tissue. Results showed that the concentration of cfDNA fragments from the patients for TISF ranged from 7.2 to 1,397 ng/ml. At least one tumor-specific mutation was identified in all 10 patients (100%). Further analysis of TISF ctDNA revealed a broad spectrum of genetic mutations, which have been reported to have clinical relevance. The analysis of concordance between TISF and tumor tissue reflected the spatiotemporal heterogeneity of glioma. Collectively, TISF ctDNA was a powerfully potential source for characterizing the genomic landscape of glioma, which provided new possibilities for precision medicine in patients with glioma.

## Introduction

Glioma is a tumor with high molecular heterogeneity, its constantly evolving genetic landscape can be a huge obstacle to the clinical management of patients (1). Studies about sequential glioma biopsies have unearthed enormous divergence of the cancer genome between the initial and recurrent tumor from the same patient (2, 3). The need for precision medicine is to characterize the real-time molecular status of the tumor, which is believed to have therapeutic significance for glioma patients (4). However, serial biopsies constitute a high risk to patients and are not realistic in clinical practice.

Liquid biopsy, defined as biofluids used for molecular diagnostics (5), has brought new hope and possibilities to precision medicine. For example, blood-based circulating tumor DNA (ctDNA) detection has proved to be of great clinical significance for a variety of solid tumors (5), such as breast (6), gastric (7), and esophageal (8) cancers. However, regarding the CNS, identifying detectable ctDNA in plasma remains challenging (9). Current studies reported that cerebrospinal fluid (CSF) could be a promising reservoir of ctDNA for brain glioma (10, 11), but with the concomitant imperfection that tumor shedding DNA to the CSF is affected by tumor burden and tumor abutting CSF (11).

The body fluid for liquid biopsy should include any conceivable stable-acquired fluid collection (12). The surgical resection for glioma always creates a cavity at the tumor site, inside exists fluid that connects or does not connect with the CSF reservoir. Here, we coined the term “Tumor *in situ* fluid (TISF)” to describe the fluid in the local surgical cavity. It is known that most gliomas relapse at the local site (13, 14), thus tumor-shed biomarkers would congregate in TISF over the process of treatment resistance and tumor progression. Moreover, with an intraoperatively implanted reservoir, obtaining TISF postoperatively is clinically feasible and uninjurious. Based on this hypothesis, we retrospectively investigated whether ctDNA from TISF could be a new source of liquid biopsy for precision medicine for patients with glioma.

## Materials and Methods

### Patients and Ethics Statement

This study included tumor *in situ* fluid (TISF) and tumor samples from 10 patients who were diagnosed as glioma (WHO grade III or IV) and treated in Henan Provincial People’s Hospital (HPPH). All patients received surgical treatment with a reservoir implanted into the surgical cavity intraoperatively and TISF were collected after surgery. The retrospective study was approved by the institutional review board and ethics committee of HPPH (Zhengzhou, China). Written informed consent was signed by all patients and/or their legal representatives.

### Sample Collection

**Figure 1:**
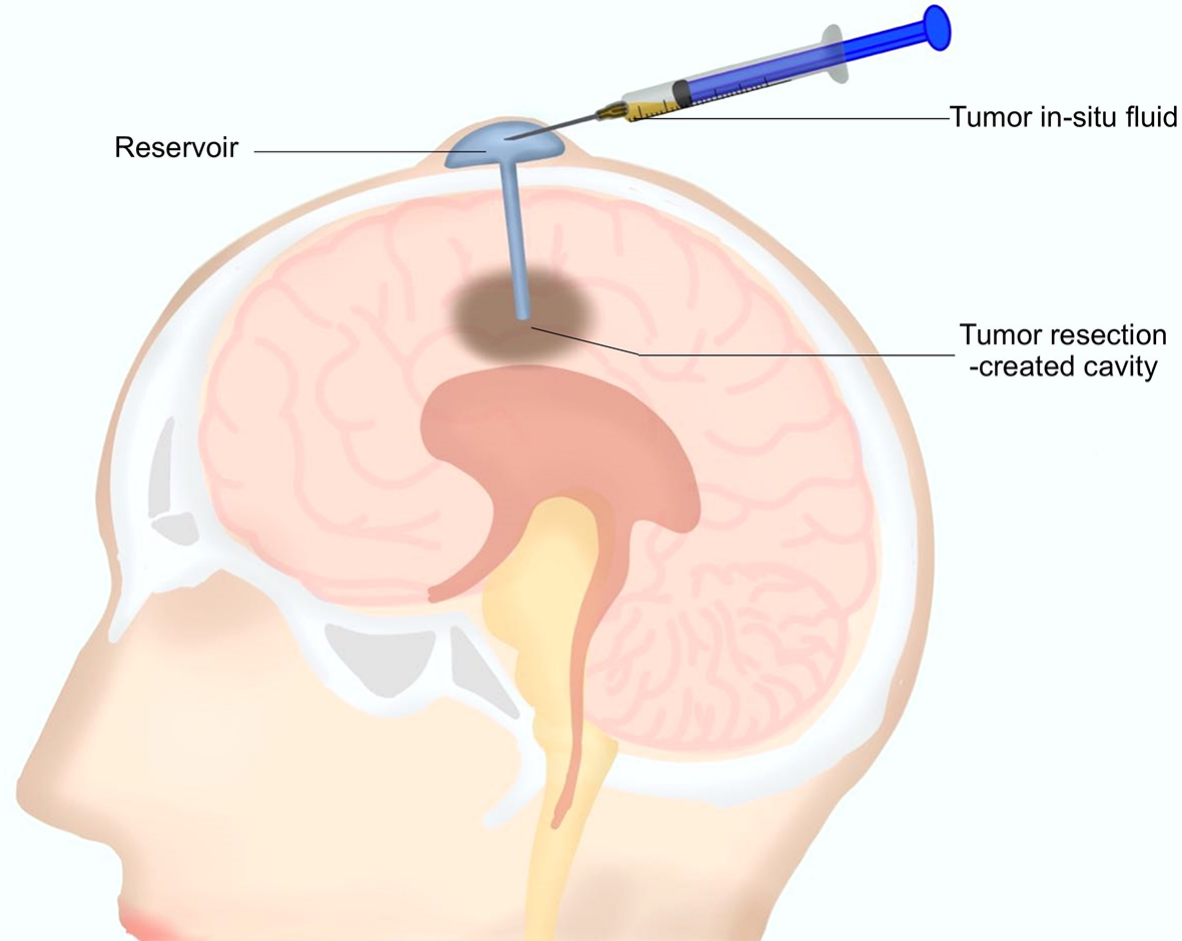
A diagram for the tumor *in situ* fluid (TISF) collection. A reservoir was implanted into the resection cavity during the resection surgery. The dome end was placed beneath the scalp attached to the catheter that was inserted within the brain leading to the tumor resection-created cavity. A small amount of fluid could be extracted with a disposable milliliter syringe from the resection cavity *via* this reservoir postoperatively.

As mentioned above, a reservoir for postoperative local chemotherapy was indwelled into the resection cavity during the surgery for every patient. After surgery, 0.5 to 1 ml TISF was collected for every patient using the indwelled reservoir (**Figure 1**). Unstained paraffin-embedded tumor tissues (UPETT) were collected, and tumor DNA was isolated for 9 of the 10 cases (90%). Blood samples (5 ml) for germline DNA control from each patient were also obtained.

### Isolation of Tumor DNA and Germline DNA

As described elsewhere (10), EDTA tubes containing biofluids (TISF or CSF or blood) were centrifuged at 1,900*g* for 10 min, after that, the supernatants from those were additionally centrifuged at 16,000*g* for 10 min. And then samples were stored at −80°C before extraction. Genomic DNA was extracted from UPETT with the QIAamp DNA Tissue & Blood Kit (Qiagen; Germantown, MD, USA). Cell-free DNA (cfDNA) was extracted from biofluids using the MagMAX™ CellFree DNA Isolation Kit (ThermoFisher Scientific; Waltham, MA, USA). Finally, all segregated DNA was quantified using the Qubit 2.0 Fluorometer with the Qubit dsDNA HS Assay kit (Life Technologies; Carlsbad, CA, USA) following the advised protocol.

### NGS Library and Sequencing Data Analysis

As described elsewhere, the DNA libraries were captured with a designed panel of 68 genes for brain tumors (GenetronHealth; Beijing, China) (10), those genes containing major brain tumor-related genes. The DNA sequencing was based on novaseq high-throughput sequencing platform.

### Statistical Analysis

The log-rank test was used to compare the survival difference between different groups, and the nonparametric Mann Whitney test was for difference analysis between the two groups.

## Result

### Patient Characteristics

**Figure 2:**
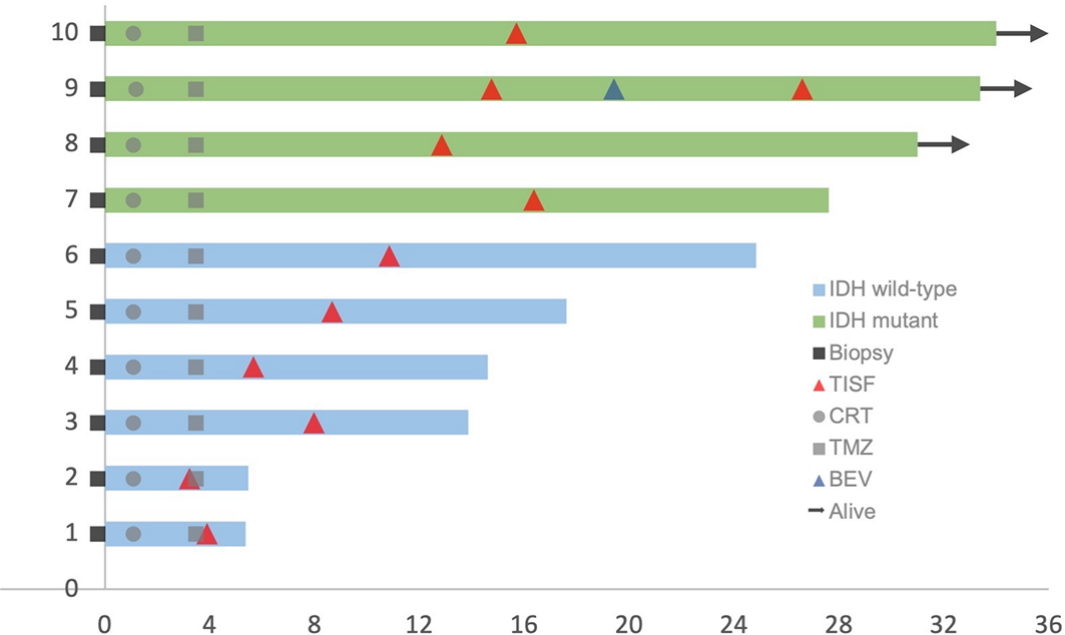
Clinical courses of included patients. Each patient began from the primary surgery (Biopsy). TISF, tumor *in situ* fluid; CRT, concurrent chemoradiotherapy; TMZ, adjuvant temozolomide chemotherapy; BEV, bevacizumab.

This cohort included seven female and three male who were diagnosed as WHO grade III or IV glioma, Specifically, six IDH wild-type glioblastoma (GBM) (60%), three IDH mutant low-grade (III) glioma and 1 IDH mutant GBM (40%) according to the 2016 WHO brain tumor classification with IDH status (15). The mean age was 52.7 years (33–66 years). Four tumors were located at the frontal lobe, three at the temporoparietal lobe, two at the temporal lobe, and the last one at the thalamus. Patients’ characteristics were summarized in **Table 1**. All patients received concurrent chemoradiotherapy (CRT) followed by adjuvant temozolomide (TMZ) combining with local Methotrexate chemotherapy after surgery. Clinical courses of patients were presented in **Figure 2**.

**Table 1:** Patients characteristics.

Patients	Sex	Age	WHO grade	Histopathology	IDH status	Tumor location	Interval since surgery (months)
1	F	45	4	GBM	wild type	thalamus	3.9
2	M	60	4	GBM	wild type	temporal	3.2
3	F	66	4	GBM	wild type	temporoparietal	8.0
4	F	55	4	GBM	wild type	frontal	5.7
5	M	52	4	GBM	wild type	temporoparietal	8.7
6	F	65	4	GBM	wild type	temporal	10.8
7	F	33	4	GBM	mutant	frontal	16.4
8	F	44	3	AO	mutant	frontal	12.8
9	F	54	3	AO	mutant	temporoparietal	14.7
10	M	53	3	AO	mutant	frontal	15.7

F, female; GBM, glioblastoma; AO, anaplastic oligodendroglioma.

### TISF ctDNA Represented the Genetic Landscape of Glioma

**Figure 3:**
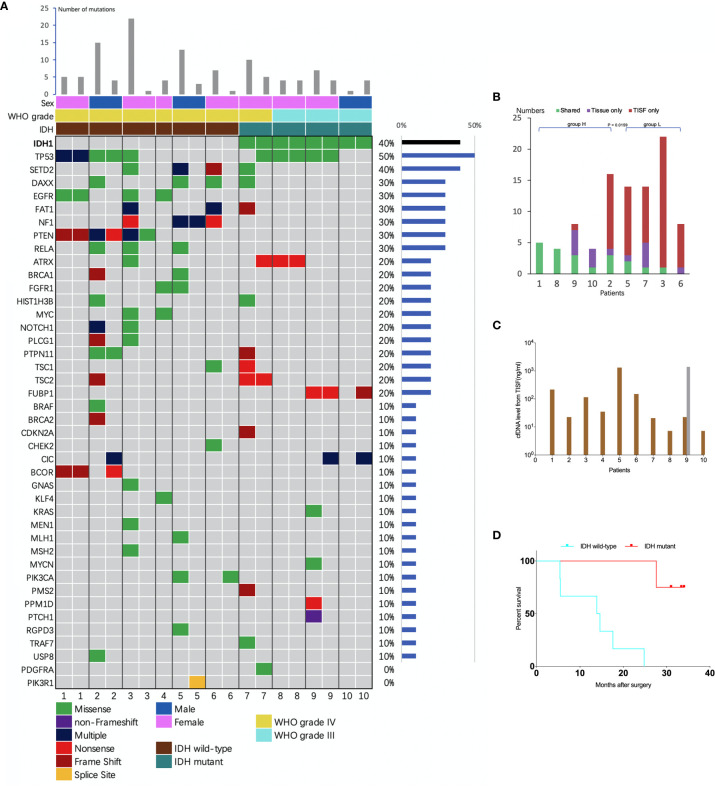
Analysis of tumor DNA from TISF and tumor tissue. **(A)** the mutational landscape for both TISF and tumor tissue. For each patient, the left column represented TISF-derived ctDNA and the right column represented tumor tissue, patient 4 just had one column for the TISF. **(B)** Concordance of tumor DNA between TISF and tumor tissue. The percentage of shared mutations varied from 0% to 100% (median 30.0%). **(C)** Cell-free DNA (cfDNA) levels from TISF. The concentration of cfDNA from TISF ranged between 7.2 and 1397 ng/ml. For patient 9, the second time TISF cfDNA experienced a significant increase. **(D)** Survival curve for patients with different IDH status. In all 10 patients (including 6 IDH wild type and 4 IDH mutant gliomas), the IDH status was accordant between the TISF and tumor tissue (100%). Median overall survival after the primary surgery was 14.2 and 32.2 months (P = 0.0038) for TISF wild-type IDH and TISF mutant IDH glioma, respectively.

The concentration of cfDNA from TISF ranged between 7.2 and 1,397 ng/ml (**Figure 3C**), which was high enough to minimize analytic errors in subsequent analysis. In total, 83 alterations of 37 different genes were detected. The average number of mutations detected in the TISF-derived ctDNA of respective individuals was 7.55 ± 6.79 (range, 1 to 22). The most frequently altered genes were TP53 (50%), SETD2 (40%), NF1 (30%), PTEN (30%), RELA (30%), DAXX (30%), EGFR (30%), and FAT1 (30%). The most common category of mutation was missense mutation (65.1%), followed by frame-shifting mutation (18.1%) and nonsense mutation (13.3%) (**Figure 3A**).

To identify whether TISF ctDNA could reflect the genome alterations of glioma, we analyzed the sequencing results in depth. Firstly, IDH status was included to determining the diagnosis for glioma in the WHO 2016 classification (15). We examined whether this genetic alteration could be detected in the TISF and matched that of the tumor tissue. In all 10 patients (including six IDH wild type and four IDH mutant gliomas), the IDH status was accordant between the TISF and tumor tissue (100%). Median overall survival after the primary surgery was 14.2 and 32.2 months for wild-type IDH and mutant IDH glioma (P = 0.0038), respectively (**Figure 3D**).

Further analysis of TISF ctDNA revealed a broad spectrum of genetic mutations which have been reported to have clinical relevance. For example, mutations of PTEN, TSC2, TSC1, NF1, and PIK3CA have been demonstrated activating PI3K/AKT/mTOR pathways, which will then boost the growth and progression of the tumor (16); FAT1 alteration is reported closely related to Wnt signaling pathway (17); modification on tumor suppressor genes, including TP53, BRCA1, BRCA2, CHEK2, and PTCH1 and on cell cycle genes, such as CDKN2A (18, 19). Many of these mutations are promising targets of precision therapeutic regimens (**Table 2**). Additionally, TMZ as an alkylating agent can induce the mutations of mis-match repair (MMR) genes, which is related to treatment resistance (20). We identified MMR gene mutations (MSH2, MLH1, and PMS2) in three patients (patients 3, 5, and 7), including two IDH wild-type GBM and one IDH-mutant GBM, all of them received systematic TMZ treatment previously, and we did not detect this signature in their tumor tissues.

**Table 2:** Potential beneficial targeted medicine for patients.

Mutations	FDA approved for this cancer	FDA approved for other cancers	Phase II/III clinical trials
CDKN2A		Palbociclib, Ribociclib	Defactinib
PIK3CA		Everolimus、Temsirolimus	Ipatasertib, GDC-0077
PTEN		Alpelisib, Copanlisib	Ipatasertib, GSK2636771
TSC1, TSC2		Everolimus, Temsirolimus	Sapanisertib, LY3023414
BRAF		Encorafenib+Binimetinib	Ulixertinib, PLX8394
BRCA2		Rucaparib, Talazoparib	
KRAS		Cobimetinib, Binimetinib	
NF1		Temsirolimus, Cobimetinib	
IDH1		Ivosidenib	
PTCH1		Vismodegi, Sonidegib	

FDA, food and durg administration. Currently, very limited medicine has been approved for treating glioma. This will be improved with the development of precision medicine.

Collectively, these results indicated that ctDNA from TISF was capable to characterize the genomic landscape of glioma during its progression process.

### Concordance Between TISF and Tumor Tissue

**Figure 4:**
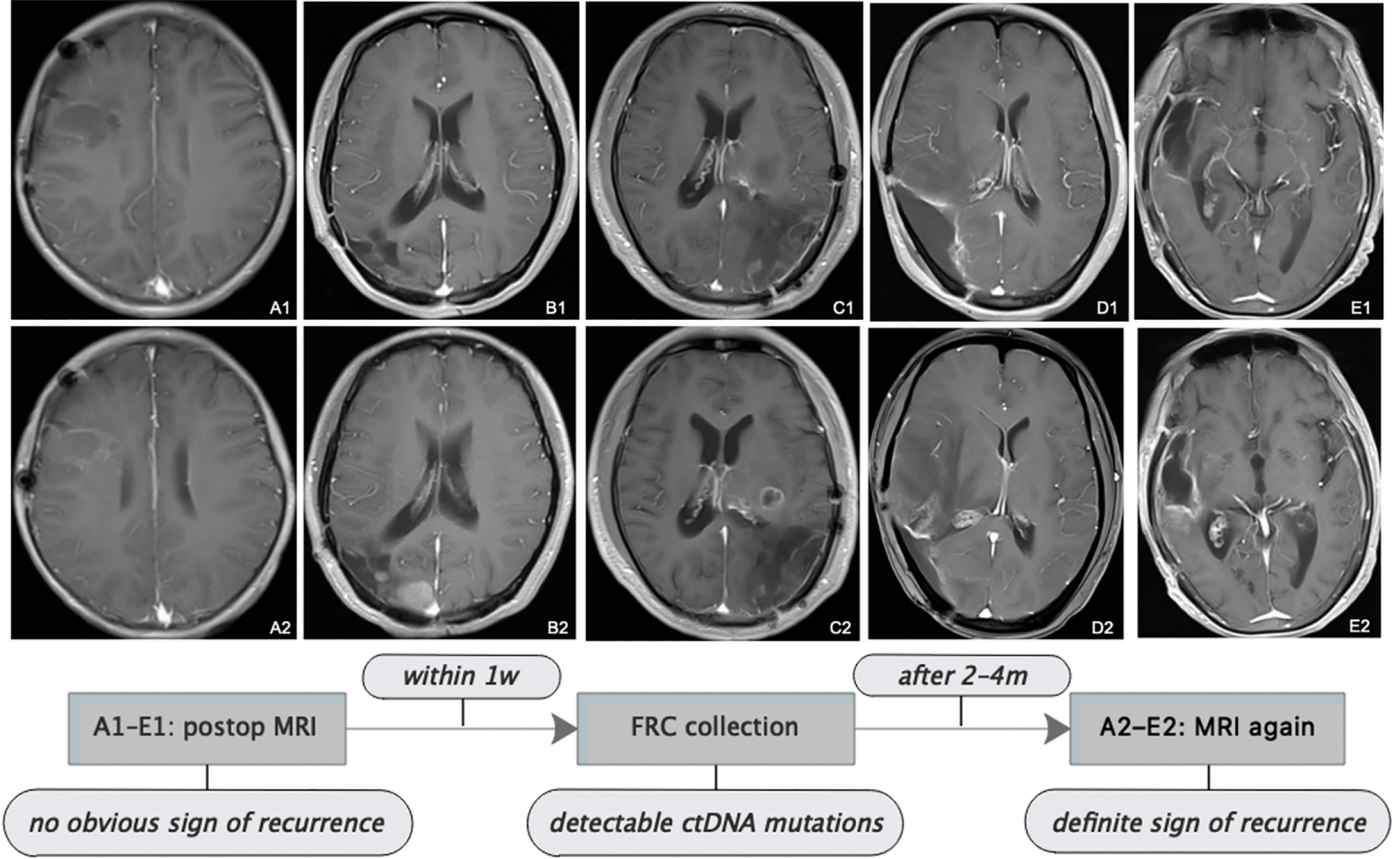
Follow-up MRI showed tumor progression. All patients received serial MRI as the standard of care. As presented in the images and the illustrative flowchart, A1–E1 stand for a postoperative representative timepoint, at which we could not notice an obvious sign of tumor progression on the MRI scans, but at which we suspected tumor progression according to the comprehensive conditions of patients, then TISF was collected, in which tumor-specific ctDNA mutations were detected. 2–4 months later, as shown in the images A2–E2, definite radiologic sign of tumor recurrence appeared. This is interesting but we still presume that the simple signature of ctDNA-positive in TISF could not be a biomarker for early predicting the recurrence of glioma, because of the special location of TISF, which is at the local site of the tumor. The tumor progression should be determined by a specific genetic analysis.

We compared the sequencing results of tumor DNA from TISF and UPETT for nine patients (**Figure 4**). The median interval between biopsy (surgery) and the first TISF collection was 10.8 months (3.2–16.4 months). The percentage of shared mutations varied from 0% to 100% (median, 31.6%) (**Figure 3B**). We further divided the 9 patients into 2 groups: group H (n=5, patients 1, 2, 8, 9, 10) included patients with low-grade glioma ((WHO grade III)) or underwent a short interval between TISF collection and tumor tissue biopsy (less than 4 months); group L included the remaining four patients (patients 3, 5, 6, 7). Subsequently, the median for the percentages of shared mutations of group H came at 54.5% (31.6%–100%), and the median for group L became 8.3% (0–25%), there was a significant difference between the two groups (P = 0.0159) (**Figure 3B**).

Despite those considerable divergences between TISF and tumor tissue, for some key genes, on the other hand, high concordance was observed between TISF and tumor tissue, one is the above-mentioned IDH1, another example is the PTEN mutation (three patients, 100%) which can activate the PI3K/AKT/mTOR signaling pathways (18). Furthermore, for patient 9 (WHO grade III anaplastic oligodendroglioma), we collected another TISF for ctDNA analysis at 26.6 months after surgery. We observed more shared mutations with tumor tissue than the first TISF (3 vs. 0) and higher accordance (60% vs. 0).

### TISF ctDNA Was Before Imaging Progression

Postoperatively, all patients had serial follow-up MRI. We noticed that TISF collection was performed at time points when apparent signs could not be spotted on the MRI scans for five patients. And it was until 2 to 4 months after TISF collection when doubtless recurrence signs could be observed on the images (**Figure 4**). However, we presumed that shedding DNA to TISF was a universal property of glioma because of the special location of TISF, thus ctDNA-positive in TISF could not be a biomarker for predicting the recurrence of glioma, and tumor progression should be determined by the specific genetic results as described above.

## Discussion

Dynamically characterizing the heterogeneity of glioma with liquid-based biomarkers is a powerful tool for precision medicine, which has been increasing the likelihood of curing malignancies (5). Here we originally demonstrated the feasibility of TISF as a steady source of liquid biopsy for glioma, providing new insight to precision medicine for brain tumors.

We identified abundant alterations derived from glioma in TISF, in-depth analysis proved that those mutations could reflect the genomic landscape of glioma during the treatment process. The landscape included PI3K/AKT/mTOR pathways-related genes, tumor suppressor genes, cell cycle genes, etc. This evidenced that multiple mechanisms contribute to the genesis and development of glioma, many of them are potential targets of precision therapies (**Table 2**).

In a previous CSF-based liquid biopsy study, Miller et al. observed a considerable divergence of shared mutations between CSF samples and tumor tissues (0–100%) across patients. Meantime, they found that samples collected at a closer interval (<3 weeks) had a higher percentage of shared mutations (79%) than those collected at a longer interval (>1,000 days) (29%) (11). Here we observed a comparable phenomenon in this pilot study. All samples were collected at a long interval (>3 months) and the percentage of shared mutations varied considerably from 0% to 100% (median, 31.6%). Interestingly, when we divided patients into two groups, i.e., group H containing patients with low-grade glioma or with a shorter interval (<4 months) and group L including the remaining patients, the fact came that group H had a higher percentage of shared mutations (median 54.5%) than group L did (median 8.3%). Several scenarios might account for these divergences. One is the spatial heterogeneity of glioma, i.e., the observation that tumor cells can show distinct genetic characteristics and one tumor biopsy usually only represents a part of tumor heterogeneity. Two is the temporal heterogeneity of glioma, which refers to that tumor can develop clonal and subclonal evolution with time, as it did in patient 9, who received two-time TISF ctDNA collection, we observed higher concordance with tumor tissue and higher cfDNA level than of the first TISF. This dynamic variation was expected correlated with the decrease in tumor burden attributed to the surgical resection and then the gradually clonal together with subclonal progression of the tumor. Another factor is due to the therapeutic pressure, which typically affects the genetic evolution of a tumor (21, 22). For example, it has been elucidated that TMZ application can induce the MMR deficit with the hypermutation ensuring in the glioma genome, which then gives contributions to the TMZ treatment resistance (20). We observed MMR gene mutations in three patients in our cohort, which was attributed to TMZ treatment. Despite those divergences and dynamics, we also noted that certain mutations were present in both tumor tissue and TISF, such as IDH1 and PTEN, which support the perspective that certain mutations or pathways occurred at the early stage of tumorigenesis and contribute to the tumor growth and progression throughout the life of the tumor (22, 23). In a word, these phenomenon and features of the tumor greatly evidenced the urgent need of precision characterization of genomic status for glioma in real time. Future frequent TISF-based liquid biopsies would be able to depict the genomic dynamics under therapeutic pressure and based on that to guide the modification of treatment combinations.

Imaging-based assessment of tumor progression for glioma cannot overcome the hysteresis and cannot reflect the molecular status of the tumor, which makes it impossible for clinicians to monitor the microscope progression of glioma from the early stage of postoperative management. In this study, we noted that detectable mutations of ctDNA from TISF were prior to clear evidence of radiologic recurrence. It was interesting but we still presumed that the simple signature of ctDNA-positive in TISF could not be a biomarker for early predicting the recurrence of glioma, because of the special location of TISF, which is at the local site of the tumor. It could be a universal property of glioma to shed tumor DNA into TISF at any stage. And we hypothesized that potential biomarkers would appear locally in high concentrations in TISF and might then be found diluted in the CSF and eventually plasma, the latter two signatures might be the adverse prognostic factors. The tumor progression should be determined by a specific genetic analysis.

In conclusion, we proved the feasibility of TISF ctDNA for characterizing the genomic landscape of glioma, this original insight provided new perspectives and possibilities for precision medicine in patients with glioma.

## Data Availability Statement

The data sets presented in this study can be found in online repositories. The names of the repository/repositories and accession number(s) can be found below: https://figshare.com/articles/dataset/original_gene_analysis_reports_zip/12672692.

## Ethics Statement

The studies involving human participants were reviewed and approved by the Institutional Review Board (IRB) and Ethics Committee of Henan Provincial People’s Hospital (Zhengzhou, China). All methods were carried out in accordance with the approved guidelines. Written informed consent was obtained from all patients. Written informed consent was obtained from the minor(s)’ legal guardian/next of kin for the publication of any potentially identifiable images or data included in this article.

## Author Contributions

XB and ZS conceived and designed the study. JY, NL, KD, and ZS collected the clinical data and samples. ZS and XB performed the data analysis. ZS wrote the manuscript. SL produced the figures. JH, HA-B, and AZ reviewed the data and revised the manuscript. YS, ZC, and ZY gave the conceptual advice. All authors contributed to the article and approved the submitted version.

## Funding

This work was supported by the Science and Technology Tackle Program of Henan Province (grant 192102310126).

## Conflict of Interest

The authors declare that the research was conducted in the absence of any commercial or financial relationships that could be construed as a potential conflict of interest.

## Abbreviations

TISF, tumor *in situ* fluid; ctDNA, circulating tumor DNA.
